# The potential role of scavenging flies as mechanical vectors of *Lagovirus europaeus*/GI.2

**DOI:** 10.1186/s12985-023-02065-4

**Published:** 2023-05-26

**Authors:** Ana M. Lopes, Tereza Almeida, Sílvia Diz, João V. Côrte-Real, Hugo C. Osório, David W. Ramilo, Maria Teresa Rebelo, Isabel Pereira da Fonseca, Pedro J. Esteves, Paulo C. Alves, Nuno Santos, Joana Abrantes

**Affiliations:** 1 CIBIO, Centro de Investigação em Biodiversidade e Recursos Genéticos, InBIO Laboratório Associado, Universidade do Porto, Campus de Vairão, 4485-661 Vairão, Portugal; 2 BIOPOLIS Program in Genomics, Biodiversity and Land Planning, CIBIO, Campus de Vairão, 4485-661 Vairão, Portugal; 3 Instituto de Ciências Biomédicas Abel Salazar (ICBAS), Unidade Multidisciplinar de Investigação Biomédica (UMIB), Universidade do Porto, Porto, 4050-313 Portugal; 4 CIISA – Centre for Interdisciplinary Research in Animal Health, Faculty of Veterinary Medicine, University of Lisbon, Lisbon, Portugal; 5 Departamento de Biologia, Faculdade de Ciências, Universidade do Porto, 4099-002 Porto, Portugal; 6 Max von Pettenkofer Institute and Gene Center, Virology, National Reference Center for Retroviruses, Faculty of Medicine, Ludwig Maximilian University of Munich (LMU) München, Munich, Germany; 7 Centro de Estudos de Vectores e Doenças Infecciosas, Instituto Nacional de Saúde Doutor Ricardo Jorge, Marateca, Portugal; 8 Instituto de Saúde Ambiental, Faculdade de Medicina da Universidade de Lisboa, Lisbon, Portugal; 9 Associate Laboratory for Animal and Veterinary Sciences (AL4AnimalS), University of Trás-s-Montes and Alto Douro (UTAD), 5000-801 Vila Real, Portugal; 10 Faculdade de Medicina Veterinária, Universidade Lusófona, Lisbon, Portugal; 11 CESAM – Centre for Environmental and Marine Studies, Faculty of Sciences, University of Lisbon, Lisbon, Portugal; 12 CITS – Center of Investigation in Health Technologies, CESPU, 4585-116 Gandra, Portugal

**Keywords:** RHDV, Flies, Vectors, Epidemiology, European rabbit

## Abstract

The European rabbit (*Oryctolagus cuniculus*) populations of the Iberian Peninsula have been severely affected by the emergence of the rabbit haemorrhagic disease virus (RHDV) *Lagovirus europaeus*/GI.2 (RHDV2/b). Bushflies and blowflies (Muscidae and Calliphoridae families, respectively) are important RHDV vectors in Oceania, but their epidemiological role is unknown in the native range of the European rabbit. In this study, scavenging flies were collected between June 2018 and February 2019 in baited traps at one site in southern Portugal, alongside a longitudinal capture-mark-recapture study of a wild European rabbit population, aiming to provide evidence of mechanical transmission of GI.2 by flies. Fly abundance, particularly from Calliphoridae and Muscidae families, peaked in October 2018 and in February 2019. By employing molecular tools, we were able to detect the presence of GI.2 in flies belonging to the families Calliphoridae, Muscidae, Fanniidae and Drosophilidae. The positive samples were detected during an RHD outbreak and absent in samples collected when no evidence of viral circulation in the local rabbit population was found. We were able to sequence a short viral genomic fragment, confirming its identity as RHDV GI.2. The results suggest that scavenging flies may act as mechanical vectors of GI.2 in the native range of the southwestern Iberian subspecies *O. cuniculus algirus*. Future studies should better assess their potential in the epidemiology of RHD and as a tool for monitoring viral circulation in the field.

## Main text

Rabbit haemorrhagic disease virus (RHDV) is a calicivirus belonging to the genus *Lagovirus* that causes rabbit haemorrhagic disease (RHD) in adult European rabbits (*Oryctolagus cuniculus*). The virus has a hepatic tropism and causes cell loss as a result of virally-induced apoptosis leading to a necrotizing hepatitis (reviewed in [1]). Originally detected in China in 1984, RHDV rapidly disseminated worldwide and reached almost all continents, becoming enzootic in several countries (reviewed in [1]). In addition to the significant ecological losses associated with the crashes in the wild rabbit populations, RHD has also led to serious negative economic impacts in the rabbit-associated industries (e.g. [2, 3]). In 2010, French rabbit populations experienced atypical RHD outbreaks [4]. These were shown to be caused by a novel RHDV genotype later named *Lagovirus europaeus*/GI.2 [5], which is antigenically dissimilar and fatally infects kittens as young as 11 days old [6]. GI.2 replaced older RHDV circulating strains (genotype GI.1; [7–10]) and quickly spread worldwide [11, 12]. Recombination is an important mechanism in GI.2 evolution with all known strains resulting from recombination events with either pathogenic (GI.1b and GII.1) or non-pathogenic strains (GI.3 and GI.4), including those associated with the first outbreaks [13–16].

Transmission of RHDV occurs via contact of a susceptible rabbit with an infected animal or carcass, through contaminated surfaces, food, burrows, cages, etc., or by vectors such as insects, scavenging birds and mammals (reviewed in [1]). Calliphorid and muscid flies were first implicated in GI.1 RHDV dissemination following the escape of the virus from a quarantine compound on the off-shore Australian Wardang Island, subsequently spreading to mainland Australia [17, 18]. Similarly, spread of RHDV to the United Kingdom was also suggested to have been mediated by insect vectors [19]. Later investigations, both in the field and under laboratory conditions, suggested that flies (*Calliphora, Chrysomya, Hydrotaea, Lucilia, Musca, Oxysarcodextia* and *Sarcophaga* genera), fleas (*Spilopsyllus cuniculi* and *Xenopsylla cunicularis*) and mosquitoes (*Aedes notoscriptus, Ae. postspiraculosus* and *Culex annulinostris*) could mechanically transmit RHDV [20–29]. Rabbits are infected by ingestion or contact with contaminated flyspots deposited on vegetation or at burrow entrances. Absorption of virus particles from flyspots deposited on mucous membranes such as rabbit conjunctiva was also put forward as a pathway of disease transmission [20]. Flyspots were shown to contain enough viral particles to cause RHD in susceptible rabbits [4]. Yet, insects do not support RHDV replication.

A recent study by Calvete and colleagues [30] showed the inability of GI.2 mechanical transmission by the mosquito *Aedes albopictus* (Culicidae) and a limited ability of the sandfly *Phlebotomus papatasi* (Psychodidae). These insects were selected for study based on their availability in laboratory colonies and their feeding habits that render them efficient mechanical vectors of viruses.

While flies were shown to be the main vectors of RHDV in Oceania [4, 15, 17, 25], their epidemiological role in Europe is unknown. In mainland Portugal, insects from orders Coleoptera and Diptera are the most commonly found near rabbit enclosures, with Psycodidae, Scarabaeidae and Staphylinidae families and *Culicoides* genus being the most abundantly trapped [31]. RHDV GI.2 has been detected in insects from Mycetophilidae, Staphylinidae and Simuliidae families, Forcipomyiinae subfamily and *Culicoides* genus [31, 32]. However, other fly species that may constitute vectors of RHDV have not been investigated. The main goals of this study were to search for evidence of mechanical transmission of GI.2 by insects collected in Portugal in the scope of a longitudinal epidemiological study of wild rabbits and to determine the potential of these insects to act as sentinels of GI.2 circulation.

## Methods

### Rabbit trapping

A longitudinal study of wild European rabbits was performed at one population in central mainland Portugal (Companhia das Lezírias, 38°50′43.7″N, 8°51′48.7″W) (Pacheco et al. 2022). Cage-traps (n = 52) were placed regularly spaced in an area of 13 hectares and baited with fresh vegetables. Eight sessions of 4–5 occasions (nights) each were performed between May 2018 and February 2019, in which 50 rabbits were captured 117 times. All rabbit specimens were identified with a subcutaneous microchip when first captured; whole blood was collected by venipuncture of the saphenous vein, placed in clotting tubes, centrifuged at 1430*g* for 10 min, and the serum was recovered and stored at − 20 °C until analysis. Rabbits were released at the site of their capture immediately after processing. Live trapping and sample collection were conducted under authorizations 580/2018, 8/2019, according to the European Union directives on the use of animals for research (Directive 2010/63/EU) and international wildlife standards [33].

The presence of RHDV GI.2-specific antibodies in rabbit sera was analyzed by an indirect enzyme-linked immune serum assay [34] with minor adaptations, as further described in [35]. Briefly, ELISA was performed by using GI.1 or GI.2 virus-like particles (VLPs) as capture antigens (100 ng/well), sera samples diluted 1/200 and a goat anti-rabbit IgG-HRP diluted 1/4,000 as conjugate. Dilutions were performed in 5% non-fat milk/PBS and incubations were carried out for 1 h at 37 °C, except for the VLPs, which were diluted in carbonate/bicarbonate buffer, pH 7.4, and incubated at 4 °C overnight. After each incubation, plates were washed 3x with PBS-0.05% Tween. Optical density was read at 450 nm within 15 min.

Any rabbit carcass found during the trapping sessions was recovered, subject to a standard necropsy procedure, and liver and lung samples collected and kept stored at − 20 °C until analysis.

### Insect trapping

From June 2018 onwards, during each rabbit trapping session and within the same site, three modified bottle flytraps [36] were set baited with rotting meat. Rabbit meat for human consumption was acquired in commercial stores, left to rot for 2–4 days at room temperature, and used as bait. Trapping lasted four days in each session in June, August, September, October, and December 2018. Trapping lasted 3 days in January 2019 and eight days in February 2019. Trapped insects were stored pooled in 70% ethanol at room temperature until analysis.

### Morphological identification

Collected flies were morphologically identified according to [37–39], Szpila (families *Calliphoridae* and *Sarcophagidae*), Grzywacz (family *Muscidae*) and sarcophagidae.myspecies.info (database with pictures: family *Sarcophagidae*), using a stereomicroscope.

Flies were pooled according to the month of collection and the morphology-based taxonomical identification. A subset of the collected flies was selected from the most abundant taxonomical units in each trapping session and subjected to molecular identification. In the peak fly abundance season, only 25% of the trapped insects were identified.

### Molecular identification

Insects were removed from the microtubes, washed in sterile water and carefully dried. Sterilized dissection material was used to separate the abdomen from the remaining parts that were stored for morphological reference. DNA and RNA were extracted from 20 to 30 mg of a pool of abdominal contents of insects from the same microtube using the AllPrep DNA/RNA Mini Kit (Qiagen) according to the manufacturer’s instructions.

For molecular identification at the genus and, whenever possible, species-level, the universal primer set for the cytochrome c oxidase subunit I gene (COI) was used [40], LCO1490: 5′-GGT CAA CAA ATC ATA AAG ATA TTG G-3′ and HCO2198: 5′-TAA ACT TCA GGG TGA CCA AAA AAT CA-3′. Reactions were performed by adding 1 µL of the extracted DNA to 5 µL of Phusion Flash High-Fidelity PCR Master Mix (Thermo Scientific), 2 pmol of each oligonucleotide and water, to a final volume of 10 µL. PCR amplification was carried out as follows: initial denaturation at 98 °C for 3 min, 40 cycles of denaturation at 98 °C for 30 s, annealing at 51 °C for 30 s and extension at 72 °C for 30 s, and a final 5 min extension at 72 °C. Amplification products with the expected size, ~ 700 base pairs (bp), were purified and sequenced on an automatic sequencer (3500xL Genetic Analyzer, Applied Biosystems) using the amplification primers. Sequences herein found were compared with those available in the GenBank database using standard nucleotide BLAST searches.

### RHDV GI.2 detection in flies

Due to the expected low viral loads in flies, a sensitive RT-qPCR method was employed for GI.2 detection (see [41] for primers and probe sequences). Amplification was performed in reactions with a final volume of 20 µL using the iTaq Universal Probes One-Step Kit (Biorad), with 1 µM and 0.2 µM of each primer and probe, respectively, 10 µL of enzyme mix and 1 µL of RNA. Cycling conditions consisted of one cycle at 10 min for 50 °C, one cycle at 95 °C for 3 min and 40 cycles of 95 °C for 15 s and 60 °C for 30 s. This system amplifies a 127 bp fragment located within the VP60 gene.

Positive RHDV GI.2 fly samples were further screened by conventional PCR. Extracted RNAs were reverse transcribed using oligo(dT) as primers and SuperScript™ III Reverse Transcriptase (Invitrogen). The primer pair RHDV6186F 5′-CAT TGA CCA CGA CAG AGG TAA C-3′ and RHDV6335R 5′-AAG GGC ACG AAC GAC ATG TCA-3′, which amplifies a fragment of 150 bp of the gene encoding the RHDV capsid protein VP60 [42–44], was used for the amplification. Reaction was performed with 1.5 µL of the cDNA reaction in a final volume of 10 µL containing 5 µL of Phusion Flash High-Fidelity PCR Master Mix (Thermo Scientific) and 2 pmol of each oligonucleotide. Cycling conditions were 3 min at 98 °C, followed by 40 cycles of 30 s at 98 °C, 30 s at 50 °C and extension at 72 °C for 10 s. Final extension was carried out for 5 min at 72 °C. Positive samples were identified by gel electrophoresis and sequenced with the amplification primers as described above.

### RHDV GI.2 detection in rabbits

RNA was extracted from the liver and lung of a single rabbit found dead in January 2019 (rabbit #118), following the protocol available from Thermo Fisher Scientific (GeneJet RNA Purification kit). In this case, RHDV detection followed a standard procedure previously developed in our lab. The use of these primer pairs allows a rapid and cost-effective detection and identification of the type of RHDV recombinant [14]. cDNA was synthesized with the NZY First-Strand cDNA Synthesis kit (NZYtech). For p16/p23, the PCR primers used were RHDV0001F 5′-GTG AAA GTT ATG GCG GCT ATG TCG-3′ and RHDV0847R 5′-CCA AGA GGA TTG ATG CAA GTG-3′ (847 bp) with the following cycling conditions: 3 min at 98 °C, followed by 40 cycles of 30 s at 98 °C, 30 s at 50 °C and extension at 72 °C for 90 s, and final extension of 5 min at 72 °C. For VP60, the PCR primers used were RHDV6186F 5′-CAT TGA CCA CGA CAG AGG TAA C-3′ and RHDV6748R 5′-CGT TAG TTG AAC CGG CCT CAG-3′ (563 bp) and the cycling conditions consisted of 3 min at 98 °C, followed by 40 cycles of 30 s at 98 °C, 30 s at 67 °C and extension at 72 °C for 30 s, and final extension of 5 min at 72 °C. In both PCRs, we used 5 µL of Phusion Flash High-Fidelity PCR Master Mix (Thermo Scientific), 2 pmol of each oligonucleotide, 1 µL of the cDNA reaction and water to a final volume of 10 µL.

## Results and discussion

**Fig. 1:**
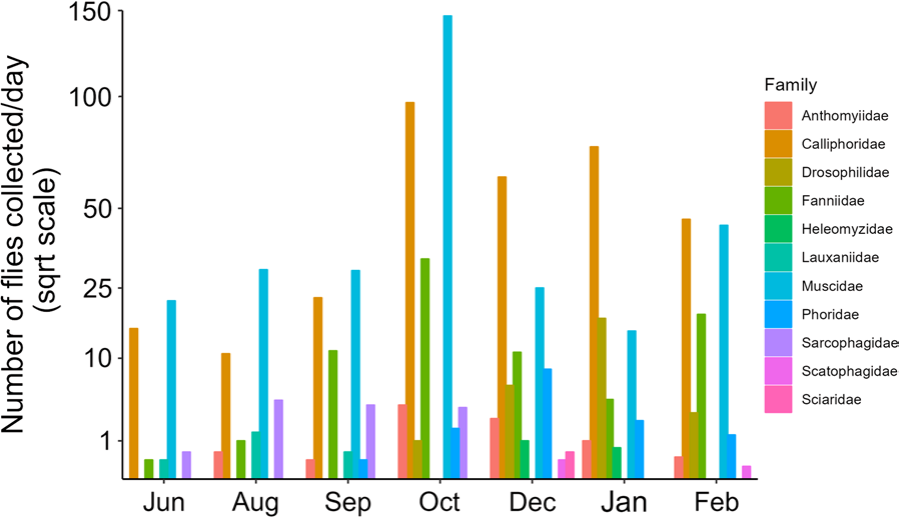
Average daily number of flies collected per trapping session. Results from the morphological identification. The Y-axis is in square root scale to improve readability

Overall, 3027 fly specimens were collected between June 2018 and February 2019 and morphologically identified. Morphological identification of each specimen was performed to the species (n = 2301), genus (n = 20), or family level (n = 706) (Table 1). The average daily number of flies collected peaked in October, with a second smaller peak in January and February (Fig. 1). The most abundant Diptera collected in every session were Muscidae and Calliphoridae specimens, as was also shown by other authors in studies carried out in the Iberian Peninsula with pig [36, 45, 46], dog [47] and cat (unpublished observations), except in January, when Drosophilidae was the second most collected family. In Portugal, *Calliphora vicina* and *C. vomitoria* can be found all year round, especially during the winter and spring seasons, while other species inside the Calliphoridae family are captured from late spring until autumn (e.g., *Chrysomya albiceps, Lucilia sericata, L. ampullacea* and *L. caesar*) [47–49]. Flies from the genus *Fannia* are also present all year round, being especially abundant during all spring season [46].

**Table 1:** Summary of the morphological identification of the collected flies by trapping session

Family	Subfamily	Genus	Species	2018					2019		Total
				Jun	Aug	Sep	Oct	Dec	Jan	Feb	
Calliphoridae	Chrysomyinae	*Chrysomya*	*albiceps*	42	28	76	16	1	0	0	163
	Luciliinae	*Lucilia*	*sericata*	8	4	7	2	0	0	0	21
			*cuprina*	0	1	0	0	0	0	0	1
	Calliphorinae	*Calliphora*	*vicina*	2	1	0	145	101	68	128	445
			*vomitoria*	0	0	0	37	22	45	79	183
Sarcophagidae	Sarcophaginae	*Ravinia*	*pernix*	0	9	4	7	0	0	0	20
		*Sarcophaga*	*argyrostoma*	0	1	0	0	0	0	0	1
			*africa*	0	2	0	0	0	0	0	2
			*tibialis*	0	1	0	3	0	0	0	4
			*portschinskyi*	0	1	1	0	0	0	0	2
			*lehmanni*	0	0	2	3	0	0	0	5
			*melanura*	0	0	0	1	0	0	0	1
	Paramacronychiinae	*Sarcophila*	*latifrons*	2	3	6	0	0	0	0	11
			*meridionalis*	0	0	2	0	0	0	0	2
Muscidae	Reinwardtiinae	*Muscina*	*prolapsa*	18	15	9	6	2	0	11	61
			*pascuorum*	7	0	0	11	0	0	4	22
			*levida*	27	49	27	506	25	7	168	809
			*stabulans*	0	1	0	0	0	0	0	1
	Muscinae	*Musca*	*domestica*	5	47	63	11	0	0	0	126
		*Eudasyphora*	sp.	0	1	0	0	1	0	0	2
	Phaoniinae	*Helina*	sp.	0	1	0	1	0	1	0	3
		*Phaonia*	sp.	0	0	1	0	1	0	3	5
	Mydaeinae	*Graphomya*	sp.	0	0	0	1	0	0	0	1
	Azeliinae	*Hydrotaea*	*ignava*	30	3	10	4	0	0	0	47
			*capensis*	0	3	8	0	0	0	0	11
			*armipes*	0	0	1	45	71	37	209	363
	Atherigoninae	*Atherigona*	sp.	0	0	0	1	0	0	0	1
Polleniidae		*Pollenia*	sp.	0	3	0	3	2	0	0	8
Fanniidae				1	4	45	132	44	13	167	406
Anthomyiidae				0	2	1	15	10	3	3	34
Scatophagidae				0	0	0	0	1	0	1	2
Lauxaniidae				1	6	2	0	0	0	0	9
Heleomyzidae				0	0	0	0	4	2	0	6
Drosophilidae				0	0	0	4	24	53	27	108
Phoridae				0	0	1	7	33	7	12	60
Sciaridae				0	0	0	0	2	0	0	2
Total				159	216	286	974	344	236	812	3027

Molecular identification by sequencing of a fragment of the COI gene (GenBank accession numbers OQ860783-OQ860806) and comparison by BLAST analysis with publicly available sequences in GenBank allowed the assignment of the insects into four families, split into 8 genera, in a total of 11 different species (Table 2). In general, there was a good agreement between morphological and molecular identification, with some instances with further assignment to the genus/species level based on the sequences obtained; yet, species identification was not possible for insects of the families Phoridae and Drosophilidae. All the detected species have been previously reported as occurring in Portugal, associated with carrion and organic matter decomposition [e.g. 36, 46, 47].

**Table 2:** Morphological and molecular identification of the insects collected, and percentage of identity with publicly available sequences

Month of collection	Sample ID	Morphological ID	Molecular ID	Consensus ID	% ID	Accession number
Jun-18	14	*Chrysomya albiceps*	*Chrysomya albiceps*	*Chrysomya albiceps*	100	MF536035
	21	*Lucilia sericata*	*Lucilia sericata*	*Lucilia sericata*	99.69	MF059331
	30	*Muscina prolapsa*	*Muscina prolapsa*	*Muscina prolapsa*	99.81	MF887459
	31	*Muscina levida*	*Muscina levida*	*Muscina levida*	98.99	MK249071
Aug-18	16	*Ravinia pernix*	*Ravinia pernix*	*Ravinia pernix*	99.69	KF038005
	18	*Muscina levida*	n.a.	*Muscina levida*	n.a.	n.a.
	20	*Muscina prolapsa*	*Muscina prolapsa*	*Muscina prolapsa*	100	KF919033
	22	*Musca domestica*	*Musca domestica*	*Musca domestica*	100	KX161463
	23	*Chrysomya albiceps*	n.a.	*Chrysomya albiceps*	n.a.	n.a.
Sep-18	25	*Muscina levida*	n.a.	*Muscina levida*	n.a.	n.a.
	28	*Musca domestica*	*Musca domestica*	*Musca domestica*	100	MN609619
	29	*Chrysomya albiceps*	*Chrysomya albiceps*	*Chrysomya albiceps*	100	MH765365
	33	Fannidae	*Fannia canicularis*	*Fannia canicularis*	99.85	MW687126
Oct-18	3	*Calliphora vomitoria*	*Calliphora vomitoria*	*Calliphora vomitoria*	100	MG969488
	4	*Calliphora vicina*	*Calliphora vicina*	*Calliphora vicina*	99.55	MT106220
	5	*Muscina levida*	n.a.	*Muscina levida*	n.a.	n.a.
	13	Fannidae	*Fannia lepida*	*Fannia lepida*	98.41	MT483682
	27	*Hydrotaea armipes*	*Hydrotaea armipes*	*Hydrotaea armipes*	100	MT920422
Dec-18	1	*Calliphora vicina*	*Calliphora vicina*	*Calliphora vicina*	99.85	JN014900
	2	*Calliphora vomitoria*	*Calliphora vomitoria*	*Calliphora vomitoria*	100	MF536037
	17	*Hydrotaea armipes*	*Hydrotaea armipes*	*Hydrotaea armipes*	99.83	KX161512
	32	*Muscina levida*	*Muscina levida*	*Muscina levida*	99.81	MK249071
	35	Phoridae	n.a.	Phoridae	n.a.	n.a.
Jan-19	6	*Calliphora vomitoria*	*Calliphora vomitoria*	*Calliphora vomitoria*	99.69	KJ394710
	11	*Calliphora vicina*	*Calliphora vicina*	*Calliphora vicina*	99.61	MG121789
	12	*Hydrotaea armipes*	*Hydrotaea armipes*	*Hydrotaea armipes*	99.65	KT082158
	34	Drosophilidae	n.a.	Drosophilidae	n.a.	n.a.
Feb-19	8	*Muscina levida*	n.a.	*Muscina levida*	n.a.	n.a.
	9	*Calliphora vomitoria*	*Calliphora vomitoria*	*Calliphora vomitoria*	99.20	MF536037
	10	*Calliphora vicina*	*Calliphora vicina*	*Calliphora vicina*	99.85	OK560160
	15	Fannidae	*Fannia lepida*	*Fannia lepida*	98.19	MT483682

*n.a.* not available

By RT-qPCR, GI.2 was detected in seven pools of insects, with Cq values ranging between 23 and 30 (Table 3). The RT-qPCR system employed in this study was previously shown to detect as few as nine copies of viral RNA, which corresponded to a mean Cq value of 37.56 [41]. Since the Cq values observed in the present study are below that value, these flies were considered as positives for the presence of GI.2. Positive insects were identified as belonging to four families, Muscidae, Calliphoridae, Fanniidae and Drosophilidae, and were collected in January and February 2019. These samples were further screened by conventional RT-PCR. Only one insect pool collected in January 2019 and identified as *Hydrotaea armipes* was positive (sample 12; Table 2). From this, we were able to sequence a 107 bp fragment within the capsid gene (GenBank accession number: OQ859633). BLAST analysis with sequences available in the GenBank database revealed the highest score with the GI.2 strain AUS/VIC/MLD-6/2017 (98.6% nucleotide identity; accession number MW460206), confirming its identity as GI.2.

**Table 3:** RT-qPCR results of the insects collected

Month of collection	Sample ID	Consensus ID	GI.2 detection
Jun-18	14	*Chrysomya albiceps*	Negative
	21	*Lucilia sericata*	Negative
	30	*Muscina prolapsa*	Negative
	31	*Muscina levida*	Negative
Aug-18	16	*Ravinia pernix*	Negative
	18	Muscina levida*	Negative
	20	*Muscina prolapsa*	Negative
	22	*Musca domestica*	Negative
	23	Chrysomya albiceps*	Negative
Sep-18	25	Muscina levida*	Negative
	28	*Musca domestica*	Negative
	29	*Chrysomya albiceps*	Negative
	33	*Fannia canicularis*	Negative
Oct-18	3	*Calliphora vomitoria*	Negative
	4	*Calliphora vicina*	Negative
	5	Muscina levida*	Negative
	13	*Fannia lepida*	Negative
	27	*Hydrotaea armipes*	Negative
Dec-18	1	*Calliphora vicina*	Negative
	2	*Calliphora vomitoria*	Negative
	17	*Hydrotaea armipes*	Negative
	32	*Muscina levida*	Negative
	35	Phoridae*	Negative
Jan-19	6	*Calliphora vomitoria*	Negative
	11	*Calliphora vicina*	Cq 27
	12	*Hydrotaea armipes*	Cq 23**
	34	Drosophilidae*	Cq 29–30
Feb-19	8	Muscina levida*	Cq 27–28
	9	*Calliphora vomitoria*	Cq 29
	10	*Calliphora vicina*	Cq 28–29
	15	*Fannia lepida*	Cq 26–27

*Based on morphological ID

**RT-PCR positive

The apparent seroprevalence of RHDV in the rabbit population was 30.0% (CI_95_ 20.5–41.5%; data not shown). No seroconversions were detected among the 50 individual rabbits trapped between May and December 2018 (117 captures), and no dead rabbits were found, supporting that RHDV was not circulating in the local population during that time. No rabbits were captured during the January-February 2019 trapping session, but the fresh carcass of an adult female, initially live-captured in August 2018 and seronegative for RHDV, was found. At necropsy, lesions suggestive of RHD were found (data not shown) and RHDV RNA was amplified from liver and lung samples. Two fragments of 762 bp and 503 bp, covering p16 and part of p23, and a partial fragment of VP60, were sequenced (GenBank accession numbers OQ859632 and OQ859631). Standard nucleotide BLAST searches revealed 97.3% and 97.6% identity with GI.2 sequences (GenBank accession numbers KM115683 and KM115716) for the p16/p23 and VP60 fragments, respectively. The GI.2 sequence obtained from the fly sample 12 (also collected in January 2019) has 100% nucleotide identity with the sequence obtained from the rabbit samples, confirming it originated from the recorded RHDV GI.2 outbreak. While identical, the samples were handled independently at different periods of time, with no possibility of cross-contamination. Moreover, the sequences obtained were not identical to any of the sequences previously obtained in our laboratory, further discarding the possibility of contamination from other tested samples.

The RT-qPCR method used in this study is a highly sensitive method that detects minimum amounts of GI.2 viral RNA [41]. Previously, Gehrmann and Kretzschmar [22] found that 10–100 virus particles were the minimum dose required to induce disease in rabbits; a more recent study showed that the minimum infective dose for GI.1 is ≤ 10^4^ gRNA copies [50]. However, this has not been fully assessed for GI.2. Thus, despite the detection of GI.2 RNA in these flies, showing their ability to carry the virus, it remains to be determined if the amount of viral particles present was sufficient to induce disease in susceptible rabbits. The detection of RHDV in the abdominal content of the flies after dissection makes it unlikely that it was the result of cross-specimen contamination in the traps or in the subsequent insect pools [24]. Thus, the virus detected would likely later be excreted in flyspots. A single flyspot (faecal and regurgitation spots) has been shown to contain enough RHDV particles to infect susceptible rabbits [20, 27].

Previous observational and experimental studies showed that muscid and calliphorid flies, including *Calliphora*, *Hydrotaea*, and *Musca*, can transmit RHD between susceptible rabbits and might have an essential role in the epidemiology [20–29]. Our results are in line with these studies as RHDV positive flies found in this study also belong to these families. We further detected GI.2 RNA in Drosophilidae and Fannidae flies, which had never been reported. The role of these flies in RHDV transmission remains to be determined; however, their life cycles and feeding habits lend support to their role as mechanical vectors for GI.2. Indeed, several RNA viruses belonging to families *Rhabdoviridae*, *Dicistroviridae*, *Birnaviridae*, *Reoviridae*, and *Errantiviridae* have been reported in *Drosophila melanogaster* [51], while *Fannia canicularis* is a known vector of Newcastle disease virus and Aleutian mink disease virus [52]. Furthermore, Fanniidae flies have a necrophagous life cycle [53], with females being attracted to decaying material, carrion and feces, as well as sweat and mucus from animals. Their larvae can also be found in vertebrate carrion and burrows, causing myasis in animals with unhealed hounds [54]. Drosophilid flies usually feed on substrates rich in bacteria, yeasts, and other fungi, but some feed on animal tissues or secretions, especially those of *Amiota, Apsiphortica* and *Phortica* genera [reviewed by 55]. Previous results reported the presence of GI.2 RNA in insects belonging to families Ceratopogonidae (genus *Culicoides* and subfamiily Forcipomyiinae), Staphylinidae, Simuliidae [31] and Mycetophilidae [32]. While their feeding habits and dispersal behaviour are compatible with a role as mechanical vectors for GI.2, their life cycles reduce the likelihood of acting as virus reservoirs [56]. However, since these insects were not detected in the present study, we could not further assess their role.

In conclusion, our results appear to indicate that scavenging flies *C. vomitoria*, *C. vicina*, *Hydrotea armipes*, *Muscina levida*, *Fannia lepida* and those from the Drosophilidae family may have a role as mechanical vectors of RHDV in the native range of the southwestern Iberian subspecies of European rabbit (*Oryctolagus cuniculus algirus*), as shown in Oceania [24, 27]. Furthermore, our observations suggest that the detection of GI.2 in scavenging flies might be used as a tool to monitor viral circulation [24]. Indeed, we were able to detect GI.2 in scavenging flies during a GI.2 outbreak, but not when there was no  evidence of viral circulation in the local rabbit population. Future studies are warranted to fully determine the potential of scavenging flies in the epidemiology of RHD and as monitoring tools for surveillance of RHDV outbreaks in the field.

## Data Availability

The datasets generated and/or analysed during the current study are available in the GenBank database under the accession numbers: OQ860783-OQ860806; OQ859631-OQ859633.

## Abbreviations

RHD: Rabbit haemorrhagic disease
RHDV: Rabbit haemorrhagic disease virus
RHDV2: Rabbit haemorrhagic disease virus 2
RHDVb: Rabbit haemorrhagic disease virus b
